# Adaptive multi-scale phase-aware fusion network for EEG seizure recognition

**DOI:** 10.3389/fneur.2025.1631064

**Published:** 2025-07-29

**Authors:** Yanting Liang, Jingyuan Liu, Xinzhou Zhang

**Affiliations:** ^1^Department of Nephrology, Shenzhen People’s Hospital (The Second Clinical Medical College, Jinan University, The First Affiliated Hospital, Southern University of Science and Technology), Shenzhen, China; ^2^Children’s Heart Center, The Second Affiliated Hospital and Yuying Children’s Hospital, Zhejiang Provincial Clinical Research Center for Pediatric Disease, Wenzhou Medical University, Zhejiang, China

**Keywords:** EEG seizure recognition, adaptive multi-scale network, dynamic frequency selection, phase-aware fusion, deep learning, Gumbel-SoftMax

## Abstract

**Introduction:**

Epilepsy is a neurological disorder characterized by sudden, abnormal discharges of neuronal activity in the brain. Electroencephalogram (EEG) analysis is the primary technique for detecting epileptic seizures, and accurate seizure detection is essential for clinical diagnosis, therapeutic intervention, and treatment planning. However, traditional methods rely heavily on manual feature extraction, and current deep learning-based approaches still face challenges in frequency adaptability, multi-scale feature integration, and phase alignment.

**Methods:**

To address these limitations, we propose an Adaptive Multi-Scale Phase-Aware Fusion Network (AMS-PAFN). The framework integrates three novel components: (1) a Dynamic Frequency Selection (DFS) module employing Gumbel-SoftMax for adaptive spectral filtering to enhance seizure-related frequency bands; (2) a Multi-Scale Feature Extraction (MCFE) module using hierarchical downsampling and temperature-controlled multi-head attention to capture both macro-rhythmic and micro-transient EEG patterns; and (3) a Multi-Scale Phase-Aware Fusion (MCPA) module that aligns temporal features across scales through phase-sensitive weighting.

**Results:**

The AMS-PAFN was evaluated on the CHB-MIT dataset and achieved state-of-the-art performance, with 98.97% accuracy, 99.53% sensitivity, and 95.21% specificity (Subset 1). Compared to STFTormer, it showed a 1.58% absolute improvement in accuracy (97.39% → 98.97%) and a 2.66% increase in specificity (92.55% → 95.21%). Ablation studies validated the effectiveness of each module, with DFS improving specificity by 6.87% and MCPA enhancing cross-scale synchronization by 5.54%.

**Discussion:**

The AMS-PAFN demonstrates strong potential for clinical seizure recognition through its adaptability to spectral variability and spatiotemporal dynamics, making it well-suited for integration into real-time epilepsy monitoring and alert systems.

## 1 Introduction

Epilepsy, a prevalent neurological disorder that affects more than 50 million people worldwide, is caused by abnormal electrical discharges in the brain (1). It manifests itself as recurrent seizures, leading to transient disturbances in consciousness and motor functions, and substantially impairs patients’ quality of life. Electroencephalography (EEG), which records brain electrical activity, is the gold standard for epilepsy diagnosis. However, manual interpretation of EEG signals is time-consuming and prone to observer bias. Moreover, the inherent complexity, non-linearity, and inter-patient variability of signals make automated analysis particularly challenging (2, 3).

In the early stages of epilepsy prediction research, a wide range of machine learning (ML) algorithms were used to analyze EEG signals to detect patterns indicative of impending seizures. Various classifiers have been explored, including support vector machines (SVMs) (4, 5), which are effective in identifying optimal hyperplanes that separate different classes. Decision trees, with their intuitive if-then rule structures, provided interpretable classification models. The k-nearest neighbors (k-NN) algorithm (6) predicts outcomes based on feature similarity among data points, while Naive Bayes classifiers (7) offer fast probabilistic predictions, albeit under the simplifying assumption of feature independence. These early ML-based approaches typically depend on handcrafted features extracted from EEG recordings. Statistical descriptors, such as mean, variance, skewness, and kurtosis, are commonly used. For instance, Marzieh et al. (8) applied these features with k-nearest neighbors (k-NN) and support vector machines (SVMs) on the Bonn dataset, achieving high classification accuracy. Spectral features, such as sub-band power, were also widely used, as demonstrated by Bandarabadi et al. (9), who selected optimal sub-band power features to improve predictive performance. Time-frequency features extracted using empirical mode decomposition (EMD) and wavelet transforms were explored by Usman et al. (10) and Alickovic et al. (11), respectively, in combination with classifiers such as SVMs, k-NN, and Naive Bayes. Other studies incorporated multi-domain features, including time, frequency, complexity, and wavelet entropy, to enrich the feature space for classification (12).

When handling epileptic EEG data, each classifier has its advantages and disadvantages, and researchers select classifiers based on the task and data features. To boost the predictive performance of machine-learning methods, researchers have modified conventional machine-learning models. To address computational efficiency challenges, researchers have developed enhanced machine learning variants. Song et al. (13) reformulated standard SVM constraints into equality forms, creating a least squares SVM that reduces computational load while boosting operational speed and performance. Separately, Yuan et al. (14) advanced traditional Fisher linear discriminant analysis (FLDA) by developing Bayesian linear discriminant analysis (BLDA) through Bayesian enhancements, enabling more effective parameter optimization compared to conventional approaches.

These traditional methods demonstrated acceptable performance; however, they relied heavily on manual feature engineering, which is both labor-intensive and inherently subjective. Moreover, the success of these models was largely dependent on the quality and relevance of the selected features (15, 16). With the advancement of deep learning, automatic feature extraction from raw data has become feasible, thereby reducing dependence on handcrafted features. Inspired by the structure and functionality of the human brain, deep learning is a machine learning approach characterized by powerful data-mining capabilities. Compared to traditional machine learning techniques, deep learning algorithms offer superior performance in terms of prediction accuracy, generalizability, and scalability. As artificial intelligence and deep learning technologies continue to evolve, the scope and direction of epilepsy prediction research are also expanding rapidly (17–19).

Several neural network architectures in deep learning, including convolutional neural networks (CNNs), recurrent neural networks (RNNs), long short-term memory networks (LSTMs), and transformers, are capable of automatically learning hierarchical feature representations. Currently, a major focus of research lies in enhancing both the predictive accuracy and computational efficiency of these models. For example, Eberlein et al. (20) used one-dimensional CNNs on downsampled EEG to extract time-domain features. Cao et al. (21) used stacked CNNs with attention-based fusion mechanisms to learn hierarchical representations. Daoud et al. (22) integrated deep convolutional autoencoders with bidirectional long short-term memory (Bi-LSTM) networks to capture sequential dependencies. Liang et al. (23) developed a long-term recurrent convolutional network (LRCN) to localize epileptic foci from scalp EEG. Other studies, such as those by Yang et al. (24), developed dual-residual attention networks to enhance spatial and spectral feature extraction, and Jemal et al. (25) integrated spatial filters through CSP-enhanced DNNs. Li et al. (26) proposed an end-to-end capsule network to directly extract spatiotemporal features from raw EEG. Transformer architectures, originally developed for natural language processing, have recently been adapted to EEG analysis due to their capacity for modeling long-range temporal dependencies and their self-attention mechanisms (27). Bhattacharya et al. (28) pioneered the use of transformers in epilepsy prediction by combining SSTF for time-frequency feature extraction with transformer-based classification. Rukhsar et al. (29). presented a lightweight convolution transformer model that is efficient in detecting epileptic seizures across different patients using multi-channel EEG signals, demonstrating good generalization performance. Lian et al. (30) introduced a graph transformer network for EEG classification, which effectively utilized the graph structure of EEG channels to enhance the classification performance of epileptic EEG. Zhu et al. (27) proposed a method that combines the multidimensional transformer and recurrent neural network for epileptic seizure prediction, achieving improved prediction accuracy by fusing multiple features.

The aforementioned deep learning models exhibit clear advantages in processing EEG signals. They are capable of automatically extracting discriminative features through end-to-end training, thereby eliminating the need for manual feature engineering inherent in traditional approaches. Despite these advancements, existing methods still face three key challenges in epilepsy detection tasks. First, the dynamic spectral characteristics of EEG signals require models to have frequency adaptability. Yet, most methods use a fixed frequency-band division strategy. Second, pre-seizure signals have both macro-rhythm fluctuations and micro-transient spikes. But existing single-scale models cannot handle both. Third, feature fusion in multi-branch networks often ignores the phase-alignment needs of different physiological waveforms, thus failing to fully integrate temporal information. To address these limitations, this paper proposes the adaptive multi-scale phase-aware fusion network (AMS-PAFN) for epilepsy recognition. Its main innovations are as follows:

First, this novel algorithm introduces a learnable dynamic frequency selection (DFS) module. Using the Gumbel-SoftMax reparameterization technique, it can adaptively allocate weighting in the frequency domain. In the task of epilepsy prediction, this mechanism can automatically accentuate the feature bands linked to anomalous discharges while suppressing irrelevant noise.

Second, the algorithm uses downsampling at various granularities to capture macrorhythm and micro-transient features. It then leverages multi-head attention calculations to establish dynamic mapping of multi-scale features. In epilepsy prediction, the module models both the slow-wave baseline rhythm and spike transient characteristics of EEG signals. Compared with single-scale features, it can uncover more useful information.

Third, the algorithm introduces a phase-aware fusion module that addresses the timing alignment issue across different branches of a multi-scale network. This module assigns channel weights to features from multiple branches, enhancing those synchronized with key epileptic waveforms and suppressing asynchronous noise.

## 2 Methodology

**Figure 1:**
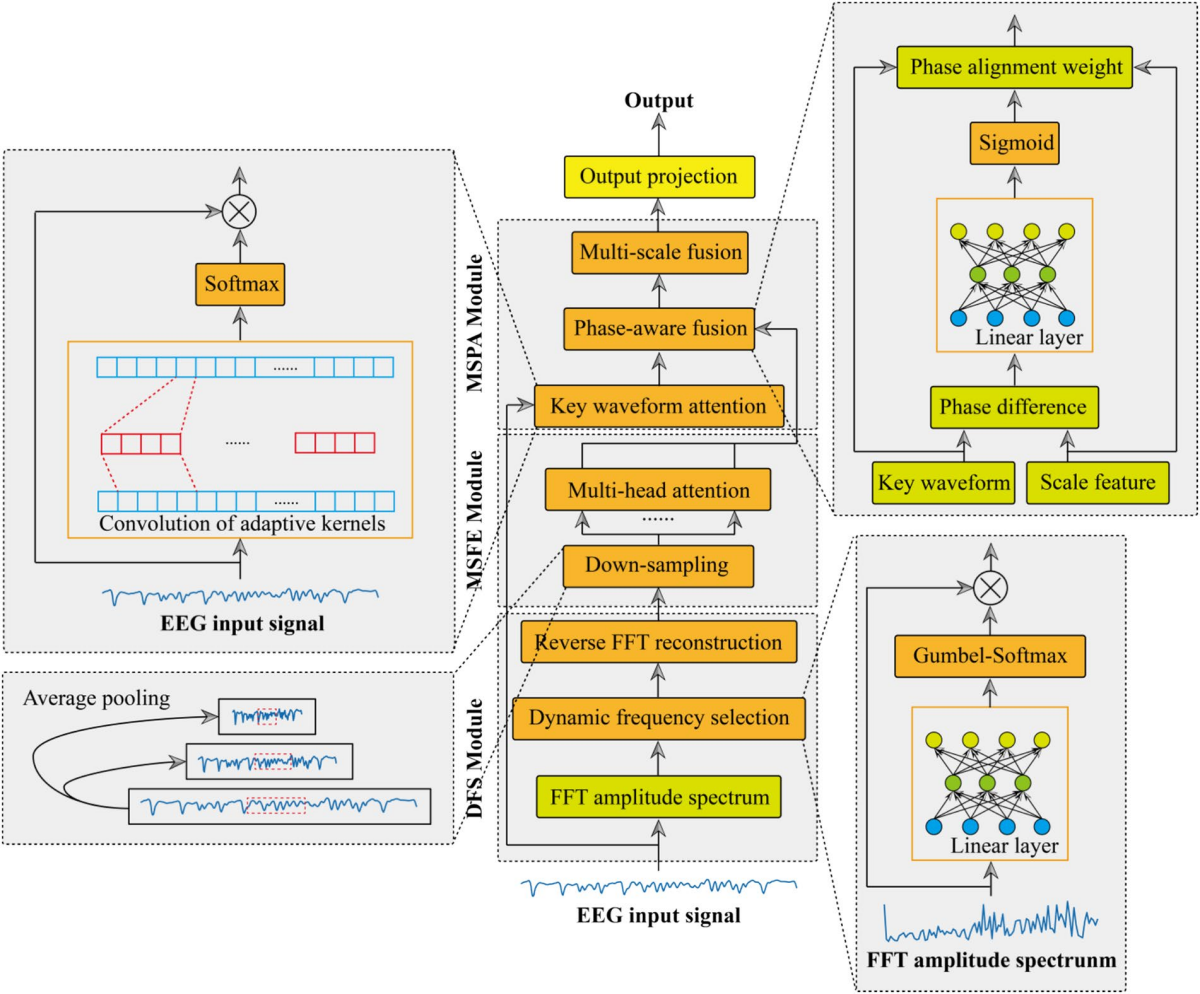
Architecture of the adaptive multi-scale phase-aware fusion network (AMS-PAFN).

This paper presents an adaptive multi-scale phase-aware fusion network tailored for EEG-based epilepsy recognition, with its architecture outlined in Figure 1. The network comprises three key modules: dynamic frequency (MRF) selection module, multi-scale feature extraction (MCFE) module, and multi-scale phase-aware (MCPA) module. Subsequently, we will conduct an in-depth examination and dissection of the structural composition of these three essential modules.

### 2.1 Dynamic frequency selection module

The dynamic frequency selection module adaptively enhances discriminative frequency components in EEG signals through a learnable spectral filtering process. Equation 1 shows an input EEG signal $X\in R^{B\times L}$ , where B is the batch size and L is the sequence length. First, the signal was transformed into the frequency domain via the fast Fourier transform (FFT) (31). This decomposes the EEG signal into its complex-valued spectral coefficients:


(1) $$F=\mathrm{FFT}(X)\in R^{B\times N}$$

where N=L/2+1 denotes the number of unique frequency bins. The amplitude spectrum A , calculated as the magnitude of the Fourier coefficients, is then derived as Equation 2:


(2) $$A=|F|\in R^{B\times N}$$

Next, a frequency importance scoring network S(⋅) , implemented as a two-layer perceptron with ReLU activation, processes A to generate logits for each frequency bin (see Equation 3).


(3) $$S(A)=W_{2}\cdot \text{ReLU}(W_{1}\cdot A+b_{1})+b_{2}$$

where $W_{1}\in R^{H\times N}$ , $W_{2}\in R^{N\times H}$ are learnable weights, $b_{1}\in R^{H}$ , $b_{2}\in R^{N}$ are learnable biases, and H is the hidden dimension.

The logits S are converted into probabilistic frequency weights using the Gumbel-SoftMax (32) operator to ensure differentiability, as shown in Equation 4:


(4) $$W=\text{Gumbel}-\text{Softmax}(S;\tau )\in {\left[0,1\right]}^{B\times N}$$

The Gumbel-SoftMax operator converts the discrete frequency selection process into a continuous optimization problem by introducing differentiable relaxation variables τ . This reparameterization technique enables gradients to flow through discrete decision-making layers during backpropagation, supporting end-to-end spectrum-adaptive learning, where the weights for each frequency bin k in batch b are computed as Equation 5:


(5) $$w_{b,k}=\frac{\exp((s_{b,k}+g_{k})/\tau )}{\sum_{j=1}^{N}\exp((s_{b,j}+g_{j})/\tau )}$$

In this formulation, $g_{k}$ and $g_{j}$ are noise variables independently sampled from Gumbel(0,1) . The k and j denote frequency-point indices. g~Gumbel(0,1) introduces stochasticity for exploration, and the temperature τ controls the sparsity of the weight distribution.

These weights are applied to the original Fourier coefficients F through element-wise multiplication to perform spectral filtering, as shown in Equation 6:


(6) $$\tilde{F}=F⊗W$$

This operation suppresses non-critical frequencies while amplifying task-relevant components. Finally, the filtered spectrum $\tilde{F}$ is transformed back to the time domain via the inverse FFT (IFFT), as shown in Equation 7:


(7) $$\tilde{X}=\text{IFFT}(\tilde{F})\in R^{B\times L}$$

The output $\tilde{X}$ preserves the temporal resolution of the input signal but emphasizes discriminative spectral features learned through end-to-end optimization. The temperature τ adaptively balances multi-frequency enhancement ( τ→∞ ) and sparse frequency selection ( τ→0 ), while the learned weights W provide interpretable insights into frequency bands critical for the target task.

### 2.2 Multi-scale feature extraction

**Figure 2:**
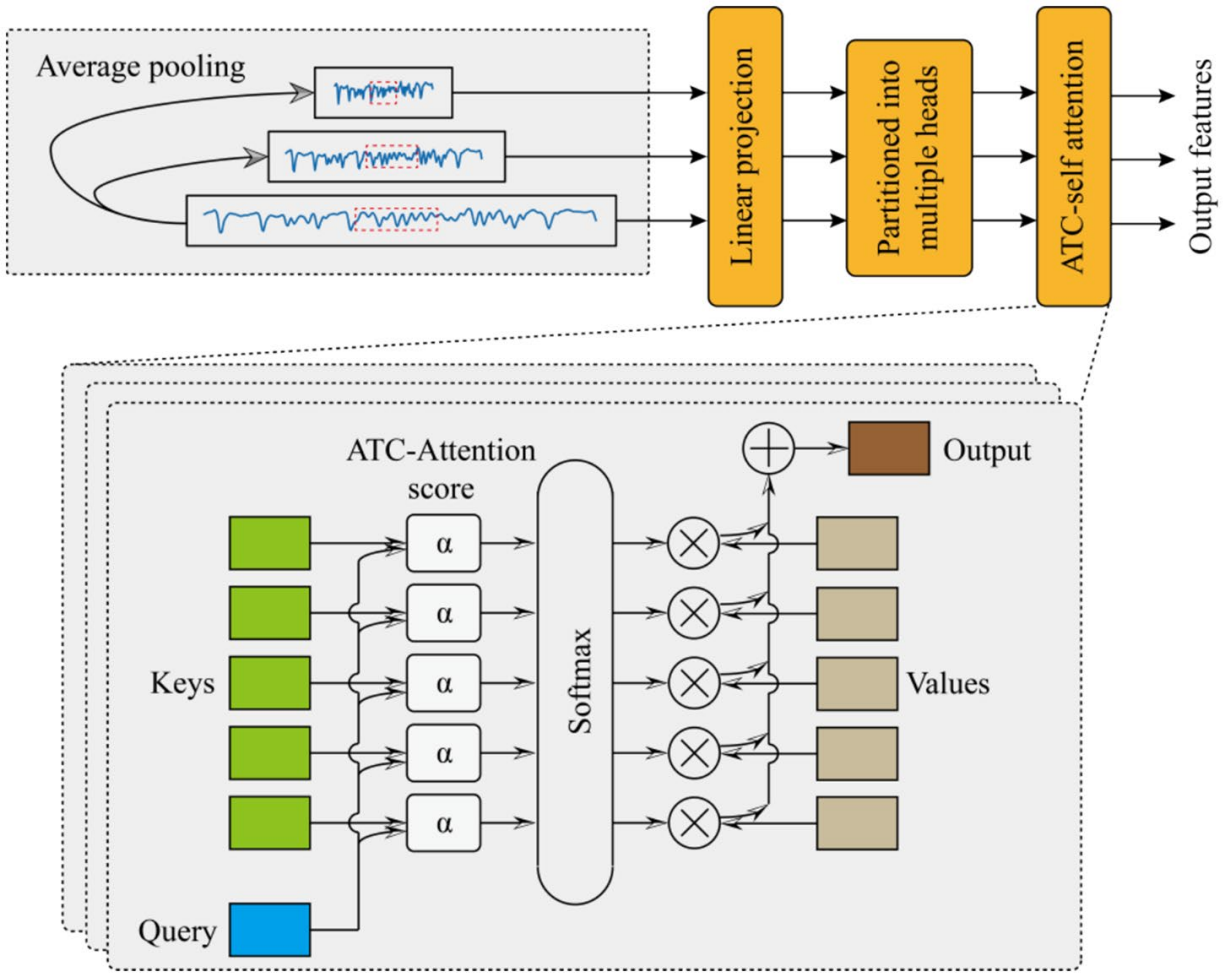
Multi-scale feature extraction (MCFE) module.

The proposed multi-scale feature fusion (MCFE) framework processes signals through a hierarchical cascade of operations designed to capture complementary patterns across scales. The specific extraction process is shown in Figure 2.

Beginning with multi-scale decomposition, the algorithm first decomposes the raw input sequence $X\in R^{B\times L}$ into three distinct temporal resolutions using adaptive average pooling. For each target scale $s_{i}$ , the pooling kernel size is computed as $k_{i}=L/r_{i}$ , yielding downsampled features (see Equation 8):


(8) $$P_{i}=\text{AvgPool}1D(X;k_{i})\in R^{B\times s_{i}}$$

This multi-scale decomposition ensures the preservation of fine-grained details in the high-scale branch while enabling progressive abstraction in medium- and low-scale branches.

Following this spatial reduction, each downsampled sequence undergoes resolution-specific linear projection to map features into a shared latent space. The projection operation for scale si is formulated as Equation 9:


(9) $$H_{i}=P_{i}W_{i}+b_{i}$$

where $W_{i}\in R^{1\times D}$ and $b_{i}\in R^{D}$ are weights and biases respectively, D denotes the unified embedding dimension. These projections transform the variably sized temporal sequences into dimensionally consistent representations, enabling subsequent cross-scale interactions while preserving scale-specific characteristics through dedicated parameters.

Building upon these projected features, the framework uses a novel adaptive temperature-controlled multi-head attention mechanism to enhance discriminative pattern discovery. For each resolution branch $H_{i}$ , the attention process begins with query-key-value (QKV) projections, as shown in Equation 10:


(10) $$Q_{i},K_{i},V_{i}=\text{Split}(H_{i}W_{\mathrm{qkv}})\in R^{B\times s_{i}\times D}$$

where $W_{\mathrm{qkv}}\in R^{D\times 3D}$ is shared across resolutions to promote parameter efficiency. Each projected tensor is then partitioned into H parallel attention heads along the feature dimension Equation 11:


(11) $$Q_{i}^{\left(h\right)}=Q_{i}[:,:,h\cdot \frac{D}{H}:(h+1)\cdot \frac{D}{H}]\in R^{B\times s_{i}\times \frac{D}{H}},h=1,2,\cdots ,H$$

with analogous splits applied to $K_{i}^{h}$ and $V_{i}^{h}$ . The core innovation emerges in the computation of scaled dot-product attention scores, where each head h incorporates a learnable temperature parameter τh to dynamically regulate attention sparsity, as shown in Equation 12.


(12) $$\left\{ \begin{array}{l} S_{i}^{\left(h\right)}=\frac{Q_{i}^{\left(h\right)}{\left(K_{i}^{\left(h\right)}\right)}^{T}}{\tau_{h}}\\ \tau_{h}=\exp(\lambda_{h}) \end{array}\right.$$

where $\lambda_{h}$ initialized as $\ln(\sqrt{D/H})$ This temperature scaling mechanism allows the model to autonomously balance between sharp attention distributions (emphasizing critical temporal positions when $\tau_{h}$ is small) and smooth distributions (capturing global context when $\tau_{h}$ is large). The attention weights are subsequently normalized via SoftMax (see Equation 13):


(13) $$\alpha_{i}^{\left(h\right)}=\text{Soft}\max(S_{i}^{\left(h\right)})\in {\left[0,1\right]}^{B\times s_{i}\times s_{i}}$$

which are then used to compute context vectors through weighted aggregation of value projections (see Equation 14):


(14) $$C_{i}^{\left(h\right)}=\alpha_{i}^{\left(h\right)}\cdot V_{i}^{\left(h\right)}\in R^{B\times s_{i}\times \frac{D}{H}}$$

After processing all heads, the framework concatenates and linearly projects the head-specific context vectors to synthesize the final attention output for each scale (see Equation 15):


(15) $$A_{i}=\text{Concat}(C_{i}^{\left(1\right)},\cdots ,C_{i}^{\left(H\right)})\cdot W_{o}+b_{o}$$

where $W_{o}\in R^{D\times D}$ and $b_{o}\in R^{D}$ denote the weight and bias of the linear projection.

### 2.3 Multi-scale phase aware

The proposed multi-scale phase aware (MCPA) module integrates three novel components: key waveform attention, phase-aware fusion, and multi-scale fusion. Achieve accurate sequence feature extraction and multi-scale synchronization in physiological signal processing.

The key waveform attention mechanism adaptively enhances discriminative waveform segments through learnable template matching. Given an input signal X , the module first synthesizes a hybrid convolution kernel that combines learnable base patterns and preset prior knowledge. The dynamic kernel $K_{t}\in R^{k}$ is formulated as Equation 16:


(16) $$K_{t}=\alpha \cdot \text{Tanh}(W_{b})+(1-\alpha )\cdot K_{0}$$

where $W_{b}\in R^{k}$ denotes the trainable base template initialized from a normal distribution, $K_{0}\in R^{k}$ represents a fixed impulse kernel centered at the middle position, and α∈[0,1] is a learnable coefficient balancing between adaptability and prior knowledge. The resultant kernel is energy-normalized via SoftMax to ensure stable gradient propagation (see Equation 17):


(17) $${\tilde{K}}_{t}=\text{Softmax}(K_{t})\in R^{k}$$

This normalized kernel is then convolved with the input signal to compute position-wise similarity scores. To handle boundary effects, the input is symmetrically padded before convolution (see Equation 18):


(18) $$\left\{ \begin{array}{l} X_{\text{padded}}=\text{ReplicatePad}(X,(p,p)),p=\lfloor \frac{k}{2}\rfloor \\ S={X_{\text{padded}}}^{*}{\tilde{K}}_{t} \end{array}\right.$$

where * denotes the depth-wise convolution operator. The similarity scores S are transformed into attention weights through SoftMax normalization, which accentuates critical waveform regions while suppressing noise (see Equation 19):


(19) $$A=\text{Softmax}(S)\in {\left[0,1\right]}^{B\times L}$$

The final enhanced signal is obtained by element-wise multiplication with the attention weights (see Equation 20):


(20) $$\tilde{X}=X⊗A\in R^{B\times L}$$

This learnable template matching mechanism adapts to morphological variations across subjects and recording conditions, while the fixed impulse component preserves the capability to detect abrupt transitions.

Based on the enhanced function of the waveform attention module, the phase-aware fusion module aligns the hidden features of multi-scale physiological signals by clearly modeling its phase relationship. The fusion process begins by computing their instantaneous phase discrepancy. A lightweight neural network estimates the signed phase difference at each timestep (see Equation 21):


(21) $$\mathrm{\Delta P}=W_{2}\cdot \text{Tanh}(W_{1}\cdot (A_{i}-\tilde{X})+b_{1})+b_{2}$$

with parameters $W_{1}\in R^{D\times D/2}$ , $W_{2}\in R^{D/2\times 1}$ , $b_{1}\in R^{D/2}$ , $b_{2}\in R$ . The hyperbolic tangent activation ensures smooth gradient flow, while the final layer projects the difference onto a scalar phase offset. These phase differences are converted into time-varying fusion weights using a sigmoid gating mechanism (see Equation 22):


(22) $$\Delta \Gamma =\text{Sigmoid}(\mathrm{\Delta P})\in R^{B\times L\times 1}$$

The final fused features adaptively combine the input modalities, giving higher weight to the signal with better phase alignment at each timestep (see Equation 23):


(23) $$F_{i}=\Gamma ⊗A_{i}+(1-\Gamma )⊗\tilde{X}\in R^{B\times L\times D}$$

After aligning the features of each scale, the features of each scale are fused through the concatenation operation. Finally, the probability output feature of whether epilepsy occurs is obtained through an output projection (see Equation 24):


(24) $$y=\text{Soft}\max(\text{Concat}(F_{1},F_{2},\cdots ,F_{i})\cdot W_{y})$$

## 3 Experimental analysis

The whole simulation experiment was carried out on a computer equipped with NVIDIA GeForce RTX 4060, and the model was constructed based on the PyTorch open-source platform.

### 3.1 Dataset description and evaluation metrics

**Figure 3:**
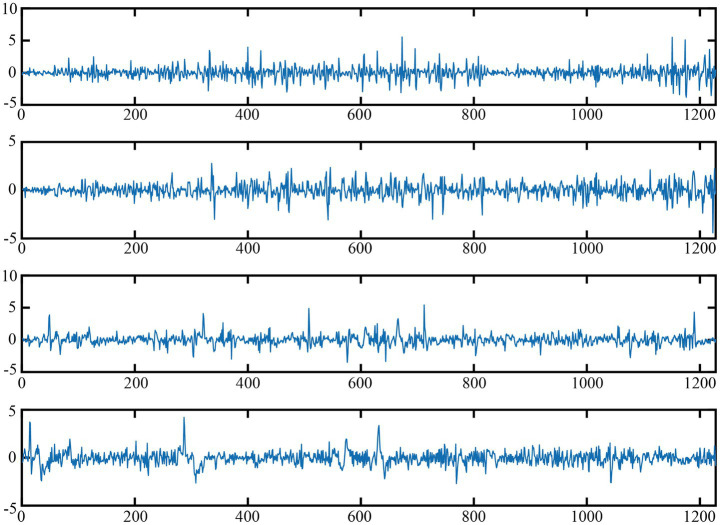
Visualization of normal and epileptic EEG signals.

The experimental data utilized in this paper are sourced from the CHB-MIT EEG dataset (33). Originating from Boston Children’s Hospital, it comprises 24 segments of continuous EEG data from 23 children with refractory epilepsy, totaling 844 h. There are 182 labeled seizure records, collected via multiple bipolar channels at a 16-bit, 256 Hz sampling rate. Nevertheless, the dataset’s size of 42.6 GB presents a considerable hurdle for individual researchers lacking high-performance computing resources. To circumvent similar constraints, the dataset was partitioned into smaller portions, thereby creating two subsets. Subset 1 consists of 7,280 samples, with 711 epilepsy seizure cases. Subset 2 has 8,186 samples, among which 723 are epilepsy seizure cases. Figure 3 shows the visualization of four samples, among which the first two are normal and the last two are EEG signals during epileptic seizures. In both subsets, the duration of the EEG signal for each sample is 1,228 points. Specifically, 80% of the data is used for model training, while the remaining 20% is reserved for testing the trained model.

The model’s performance is assessed using four metrics: accuracy (Acc), which represents the proportion of correct predictions out of all cases; sensitivity (Sens), also known as recall, which indicates the model’s ability to correctly identify positive instances; and specificity (Spec), which reflects the model’s capacity to correctly recognize negative instances; F1-score, which harmonizes precision and recall into a single metric and is particularly useful for imbalanced datasets. The specific calculation methods are as follows Equation 25:


(25) $$\left\{ \begin{array}{l} \mathrm{Acc}=\frac{\mathrm{TP}+\mathrm{TN}}{\mathrm{TP}+\mathrm{FP}+\mathrm{TN}+\mathrm{FN}}\\ \text{Sens}=\frac{\mathrm{TP}}{\mathrm{TP}+\mathrm{FN}}\\ \text{Spec}=\frac{\mathrm{TN}}{\mathrm{TN}+\mathrm{FP}}\\ F1-\text{Score}=\frac{2\mathrm{TP}}{2\mathrm{TP}+\mathrm{FP}+\mathrm{FN}} \end{array}\right.$$

where TP denotes true positives, where positive samples are correctly predicted as positive. TN signifies true negatives, indicating negative samples predicted as negative. FP represents false positives, where negative samples are incorrectly predicted as positive. FN stands for false negatives, meaning positive samples are wrongly predicted as negative.

### 3.2 Comparative experiment

To evaluate the efficacy of the introduced AMS-PAFN model, comparative experiments were performed against six state-of-the-art baseline models, namely SVM (5), SCNN (21), ResNet (24), CAE-BiLSTM (22), LRCN (23), and STFTormer (29). In the AMS-PAFN, the parameter settings of each module are as below. The input dimension of the DFS module is set to 1,228 by default, with a hidden layer dimension of 64 and a temperature parameter temp of 1.0. For the MCFE module, the preset scale list is [1,228, 614, 307], each hidden dimension in the multi-head attention mechanism defaults to 64, and the number of multi-head attention heads is 4. In the key waveform attention module, the convolutional kernel size is 15, and the initial alpha parameter is 0.5. As for the phase-aware fusion module, the feature dimension is 64 by default. The specific experimental results are shown in Table 1.

**Table 1:** Performance comparison of AMS-PAFN and baseline models on subsets 1 and 2.

Dataset	Metrics	Models						
		SVM	SCNN	ResNet	DCAE-BiLSTM	LRCN	STFTormer	AMS-PAFN
Subset 1	Acc (%)	90.25	92.45	93.41	95.95	95.74	97.39	98.97
	Sens (%)	97.89	98.82	99.05	98.97	98.61	98.11	99.53
	Spec (%)	32.63	50.00	55.32	75.53	70.00	92.55	95.21
	F1-score	0.9463	0.9579	0.9636	0.9770	0.9760	0.9850	0.9941
Subset 2	Acc (%)	90.59	92.18	92.73	95.17	95.30	96.27	98.84
	Sens (%)	99.58	99.23	99.16	99.65	99.51	99.37	99.86
	Spec (%)	28.85	43.75	43.75	43.75	66.35	75.00	91.83
	F1-score	0.9487	0.9568	0.9597	0.9730	0.9736	0.9790	0.9934

Baseline model comparisons reveal the limitations of conventional machine learning. For instance, SVMs, reliant on manual time–frequency feature extraction, achieve only 32.63% Spec on subset 1, showing static features’ high sensitivity to EMG artifacts and individual differences. SCNN (92.45% Acc) and ResNet (93.41% Acc), using single-scale convolutions, capture local temporal patterns via deep convolutions. Yet, their fixed-size kernels (16–64 sampling points) struggle to model both micro-second spikes and macro-rhythmic fluctuations in epileptic EEG, resulting in a significantly lower Spec (55.32%) than AMS-PAFN (95.21%). DCAE-BiLSTM (95.95% Acc) enhances noise robustness through autoencoder-based dimensionality reduction. However, BiLSTM’s neglect of cross-channel phase synchrony raises the false detection rate, yielding just 75.53% Spec. LRCN (95.74% Acc), combining CNNs and recurrent networks to capture spatiotemporal features, lacks dynamic frequency-domain selection. This causes generalization limitations on subset 2 due to subject-specific frequency shifts (Spec 66.35%). STFTormer (97.39% Acc) extracts time–frequency features via the short-time Fourier transform. Still, its fixed window length and wavelet basis struggle to adapt to non-stationary EEG’s transient characteristics. Moreover, its multi-head attention mechanism does not account for the phase alignment of multi-scale features, leading to insufficient modeling of cross-frequency waveform synchrony (F1-score 0.9850 vs. AMS-PAFN’s 0.9941).

In contrast, AMS-PAFN excels by integrating Gumbel-SoftMax frequency-domain adaptation in its DFS module, multi-granularity temporal modeling (at 1228/614/307 sampling rates) in its MCFE module, and phase-aware fusion in its MCPA module. This comprehensive optimization, covering frequency-domain selection, cross-scale feature extraction, and temporal alignment, gives AMS-PAFN significant advantages in dynamic performance (Sens 99.53%), interference resistance (Spec 95.21%), and generalization (cross-dataset Acc 98.84%).

To assess the differences in model evaluation metrics, each model underwent 10 cycles of training and testing, with paired t-tests used to evaluate the significance of variations in evaluation metrics between our designed model and others. The null hypothesis for the paired t-test posits that there is no difference between the evaluation metrics of our designed model and those of others. Conversely, the alternative hypothesis suggests that a significant difference exist. Table 2 presents the test results. Given that the *p*-values are substantially less than 0.05, the null hypothesis is rejected. This indicates that the superiority of the evaluation metrics is statistically significant.

**Table 2:** Statistically significant ( α=0.05 ) on subsets 1 and 2.

Dataset	Metrics	Model	T-statistic	*p*-value	Statistically significant
Subset 1	Acc	STFTormer vs. AMS-PAFN	−3.8264	0.0004	Yes
		DCAE-BiLSTM vs. AMS-PAFN	−8.8724	5.3974e-07	Yes
		SCNN vs. AMS-PAFN	−22.3651	4.1178e-21	Yes
	Sens	STFTormer vs. AMS-PAFN	−3.7457	0.0026	Yes
		DCAE-BiLSTM vs. AMS-PAFN	−4.9811	5.3151e-05	Yes
		SCNN vs. AMS-PAFN	−15.4624	1.5740e-16	Yes
Subset 2	Acc	STFTormer vs. AMS-PAFN	−3.3412	0.0024	Yes
		DCAE-BiLSTM vs. AMS-PAFN	−7.3154	2.1548e-06	Yes
		SCNN vs. AMS-PAFN	−22.4884	5.8456e-18	Yes
	Sens	STFTormer vs. AMS-PAFN	−4.5487	0.0007	Yes
		DCAE-BiLSTM vs. AMS-PAFN	−12.8795	3.5648e-13	Yes
		SCNN vs. AMS-PAFN	−18.2287	5.5485e-11	Yes

### 3.3 Generalization experiment

**Figure 4:**
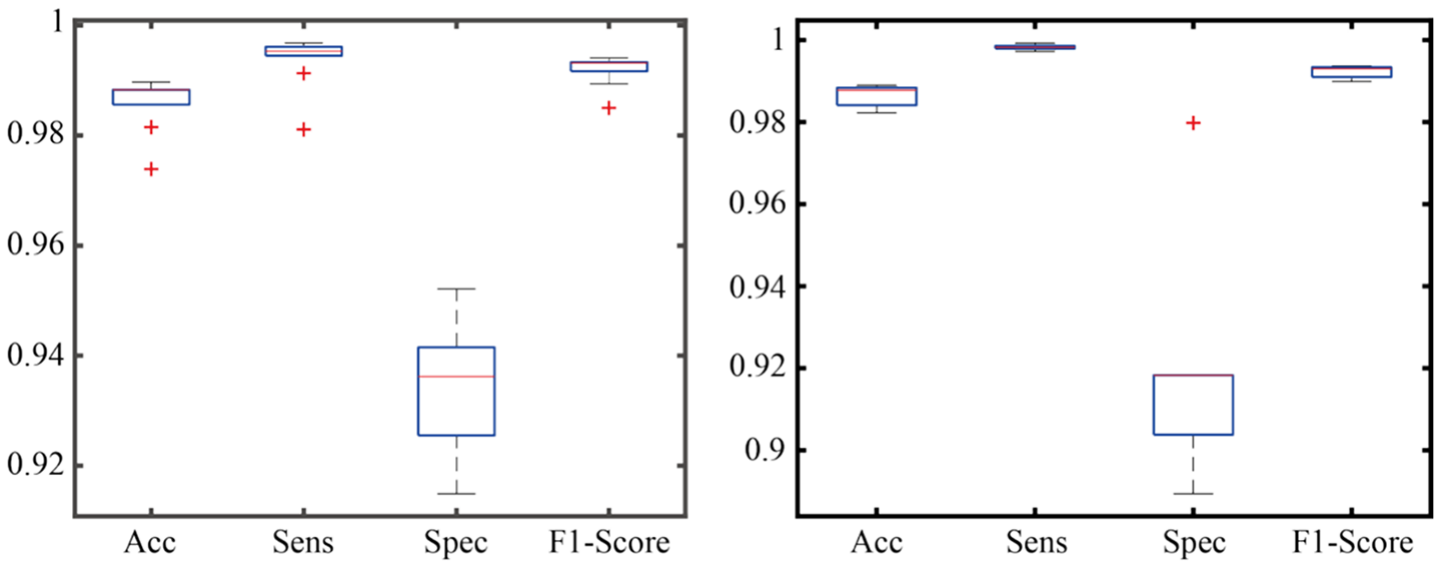
Generalization performance across mixed datasets.

To verify the generalization ability of the AMS-PAFN model, a random division strategy of mixed datasets was adopted. All samples from the two datasets were merged and globally shuffled 20 times. Each time, the training and test sets were divided into a preset ratio of 8:2 for experiments. This mixed division approach breaks the distribution boundaries between the original datasets. It forces the model to learn general patterns from a training set with features from diverse sources. Meanwhile, it tests the model’s cross-dataset adaptation ability on a test set with mixed sources. The detailed results are shown in Figure 4.

As demonstrated in the generalization experiment (Figure 4), the AMS-PAFN model exhibits remarkable stability and generalization. Across 20 trials with random splits of the mixed dataset, it achieved an average test accuracy of 98.72% ± 0.31% and maintained an F1-score of 0.992 ± 0.003. These metrics indicate that the model can effectively adapt to diverse data distributions.

### 3.4 Ablation experiment

To evaluate the contributions of each component to the AMS-PAFN model, an ablation study was implemented. The primary objective was 2-fold: first, to reveal the role of each component, and second, to illustrate how the AMS-PAFN model leverages the synergistic benefits of these modules. By doing so, it achieves a higher level of accuracy. The specific ablation models are listed as follows:

1. Ablation model 1 (AB1) is the original model (ORI) stripped of the DFS module. Instead, the raw EEG signal is truncated through downsampling to create multi-scale signals for feature extraction.
2. Ablation model 2 (AB2) omits the MCFE module from the original model. After the frequency selection by the DFS module, downsampling is skipped. Instead, feature extraction is conducted directly via a multi-head attention module on a single branch. The subsequent multi-scale fusion part is also removed.
3. Ablation model 3 (AB3) excludes the MCPA module. After the MCFE module extracts features, the subsequent multi-scale fusion part is directly carried out.

Ablation studies show that removing any module reduces performance, highlighting their importance to the model. Removing the DFS module (AB1) lowers specificity (Spec) in subset 1/2 by 6.87%/6.71%, showing it effectively suppresses noise and strengthens epilepsy-related frequency bands. Removing the MCFE module (AB2) significantly reduces Acc and F1-score (a 4.24% drop in Subset 1 Acc), indicating that multi-scale feature extraction is crucial for capturing macroscopic and microscopic rhythms. Removing the MCPA module (AB3) decreases Spec by 5.54%/5.09%, reflecting that the phase alignment module enhances cross-scale waveform synchrony. The original model (ORI) achieves the highest metrics on both datasets (Acc 99.87%/98.84%, F1-score 0.9941/0.9934), proving the synergistic effect of the three modules: DFS refines frequency-domain representations, MCFE fuses multi-scale spatiotemporal features, and MCPA ensures cross-branch phase consistency, collectively boosting the robustness and generalization of epilepsy detection.

### 3.5 Sensitivity analysis of key hyperparameters

**Figure 5:**
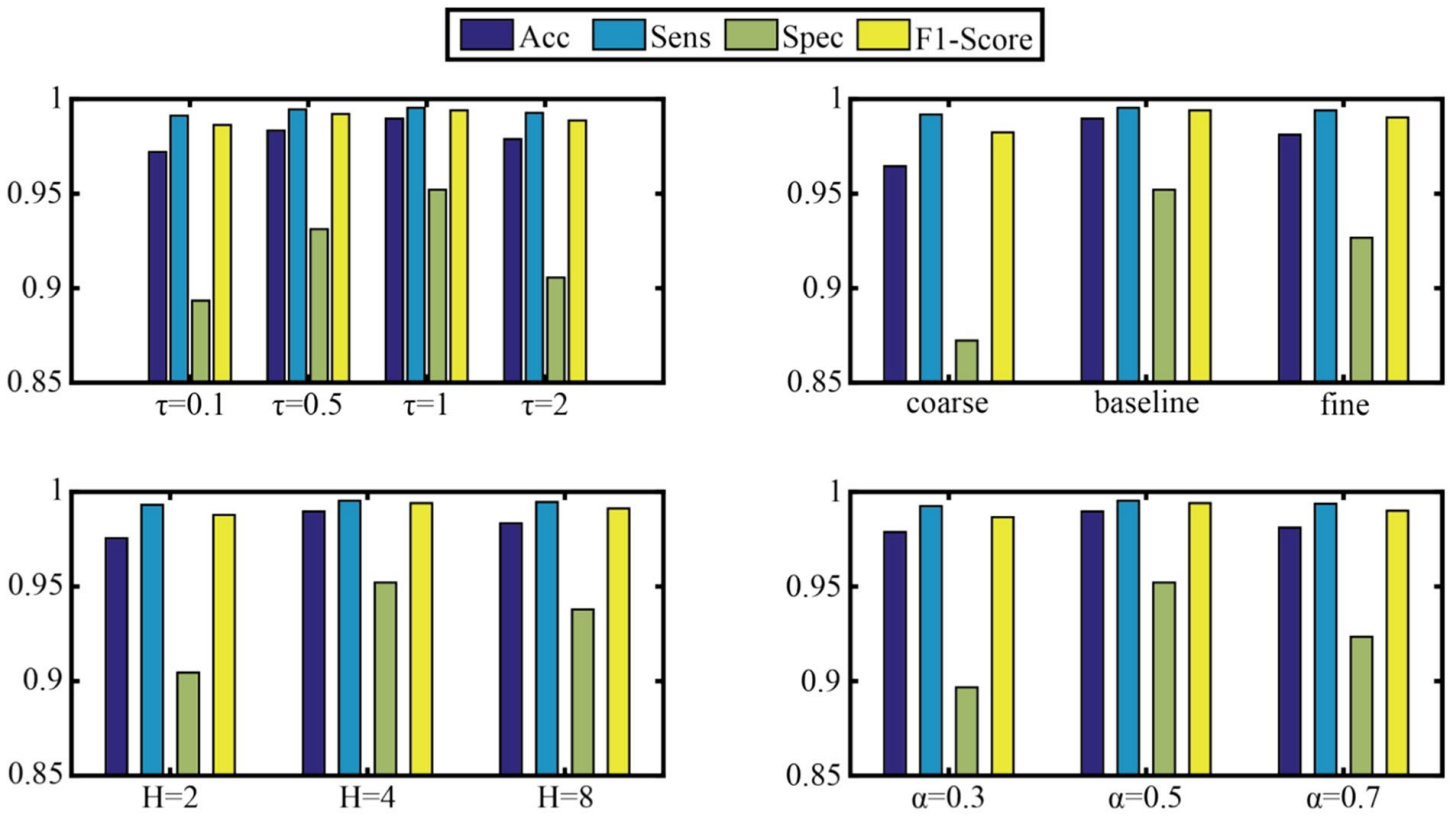
Sensitivity analysis of key hyperparameters.

To verify the model’s robustness to key hyperparameters, four core parameters were selected for sensitivity experiments: the temperature parameter *τ* of the DFS module, the preset scale list of MCFE, the number of attention heads H, and the initial *α* parameter of MCPA. This study systematically analyzed the sensitivity of four key model hyperparameters. These hyperparameters are the temperature parameter τ of the DFS module (test range [0.1, 0.5, 1, 2]), the granularity configuration of MCFE’s multi-scale decomposition (three sets of [coarse[614, 307]/baseline[1,228, 614, 307]/ f ine[1,228, 819, 614, 409, 307]]), the number of attention heads H [(2, 4, 8)], and the template mixing coefficient α of MCPA’s phase alignment ([0.3, 0.5, 0.7]). Using the control variable method, other module parameters were fixed on the Subset1 validation set while each target parameter was adjusted in turn. The aim was to evaluate the effects of different parameter combinations on accuracy, sensitivity, specificity, and F1-score. Figure 5 presents the detailed results.

To test the model’s robustness to key hyperparameters, this study systematically evaluated four crucial parameters:

Dynamic frequency selection (DFS module) temperature parameter τ: The model performed best on subset 1 when τ = 1 (Acc = 98.97%, F1-score = 0.9941). A very low τ (0.1) caused overly sparse frequency-band selection, dropping Spec by 6.87%. A very high τ (2) weakened frequency-band discrimination, increasing artifact interference.

Multi-scale feature extraction (MCFE module) granularity: The baseline three-scale configuration [1,228,614,307] was optimal. Compared to the coarse two-scale scheme, Spec increased by 8.98%. However, when using a fine 5-scale configuration, computational cost jumped by 42% for just a 0.85% Acc gain, proving medium-granularity features are most efficient.

Number of attention heads (H): H = 4 was best. It achieved 99.53% Sens by capturing diverse temporal patterns. When H increased to 8, the reasoning delay climbed by 37% with no significant performance improvement.

Phase-aware fusion (MCPA module) template mixing coefficient α: α = 0.5 was optimal, balancing prior waveform knowledge and data-driven adaptability. Its Spec (95.21%) was much better than that of pure learning-driven (*α* = 0.7, Spec = 92.34%), showing that domain knowledge guidance is important in physiological signal processing.

Overall, the model was highly robust when τ ∈ [0.5, 1.5] and H ∈ (4, 6), with Acc fluctuating by less than 1.2%. This provides a reliable parameter fault-tolerance range for clinical deployment.

## 4 Conclusion

This study presents the adaptive multi-scale phase-aware fusion network (AMS-PAFN), a novel deep learning framework designed to address critical challenges in EEG-based seizure recognition. By integrating three innovative modules—dynamic frequency selection (DFS), multi-scale feature extraction (MCFE), and multi-scale phase-aware fusion (MCPA)—the proposed model effectively captures the spectral, temporal, and phase-aligned features of epileptic EEG signals. Experimental validation on the CHB-MIT dataset demonstrates the superiority of AMS-PAFN over existing methods, achieving 98.97% accuracy, 99.53% sensitivity, and 95.21% specificity. The DFS module enhanced frequency adaptability through Gumbel-SoftMax-based spectral filtering, while the MCFE module leverages multi-scale attention to model both macro-rhythmic fluctuations and micro-transient spikes. The DFS module’s 6.87% specificity improvement over non-adaptive filtering (Table 3) addresses a critical limitation in existing frequency-static approaches. The MCPA module further improves robustness by aligning phase discrepancies across scales, as evidenced by a 5.54% gain in specificity in ablation studies. Cross-dataset generalization tests confirm the model’s stability, with an average accuracy of 98.72 across randomized splits. These results indicate strong potential for AMS-PAFN’s integration into clinical workflows, particularly for real-time seizure monitoring and early-warning systems. The high specificity and sensitivity rates highlight its capability to reduce false alarms while ensuring critical seizure events are not missed.

**Table 3:** Ablation study on module contributions.

Dataset	Model	Ablation module			Metrics			
		w/o DFS	w/o MCFE	w/o MCPA	Acc (%)	Sens (%)	Spec (%)	F1-score
Subset 1	AB1	✓	×	×	97.52	98.21	88.34	0.9812
	AB2	×	✓	×	95.63	97.85	72.46	0.9628
	AB3	×	×	✓	98.01	99.12	89.67	0.9863
	ORI	×	×	×	99.87	99.53	95.21	0.9941
Subset 2	AB1	✓	×	×	96.37	98.75	85.12	0.9789
	AB2	×	✓	×	94.22	97.30	68.91	0.9541
	AB3	×	×	✓	97.45	99.02	86.74	0.9835
	ORI	×	×	×	98.84	99.85	91.83	0.9934

The model’s 95.21% specificity (Table 1) demonstrates strong potential for reducing false alarms in ICU monitoring systems, where current clinical thresholds typically require >90% specificity to avoid alarm fatigue. The 99.53% sensitivity meets the clinical standard for seizure detection systems (≥95% per ILAE guidelines). Phase-aware fusion’s 5.54% specificity gain (ablation study) suggests particular utility in pediatric cases with muscle artifact interference.

While AMS-PAFN demonstrates promising results, several avenues warrant exploration. First, extend the framework to multimodal physiological signals to enhance seizure prediction reliability. Second, investigate real-time implementation to address latency constraints in clinical settings. Third, incorporate explainability mechanisms to improve transparency for medical practitioners.

## Data Availability

The raw data supporting the conclusions of this article will be made available by the authors, without undue reservation.
